# Competencia cultural en estudiantes de enfermería de una Universidad Pública de Colombia*

**DOI:** 10.15649/cuidarte.2779

**Published:** 2023-09-08

**Authors:** Francy Lizeth Sequeda-Villarraga, Maria Natalia Ureña-Parra, Martha Ligia Velandia-Galvis, Gloria Esperanza Zambrano-Plata

**Affiliations:** 1 Enfermera profesional. Universidad Francisco de Paula Santander, Cúcuta, Colombia. E-mail: francysequeda@gmail.com Universidad Francisco de Paula Santander Universidad Francisco de Paula Santander Cúcuta Colombia francysequeda@gmail.com; 2 Enfermera profesional. Universidad Francisco de Paula Santander, Cúcuta, Colombia. Autor de correspondencia. E-mail: marianataliaup@ufps.edu.co Universidad Francisco de Paula Santander Universidad Francisco de Paula Santander Cúcuta Colombia marianataliaup@ufps.edu.co; 3 Enfermera. Esp. en cuidado crítico del adulto. Docente Universidad Francisco de Paula Santander, Cúcuta, Colombia. E-mail: marthaligiavg@ufps.edu.co Universidad Francisco de Paula Santander Universidad Francisco de Paula Santander Cúcuta Colombia; 4 Enfermera, PhD en educación. Docente Universidad Francisco de Paula Santander, Cúcuta, Colombia. E-mail: gloriaesperanzazp@ufps.edu.co Universidad Francisco de Paula Santander Universidad Francisco de Paula Santander Cúcuta Colombia gloriaesperanzazp@ufps.edu.co

**Keywords:** Competencia cultural, Formación profesional, Diversidad cultural, Estudiantes de enfermería, Culturally Competent, Professional Training, Cultural Diversity, Students, nursing, Competencia Cultural, Capacitagao Profissional, Diversidade Cultural, Estudantes de Enfermagem

## Abstract

**Introducción:**

La diversidad representa un desafío en la atención en salud, pues supone que los enfermeros sean culturalmente competentes, capaces de brindar cuidados que se adapten a las creencias, valores y prácticas de los pacientes provenientes de diversas culturas.

**Objetivo:**

Determinar el nivel de competencia cultural en los estudiantes de enfermería de una Universidad Pública de Colombia.

**Materiales y métodos:**

Estudio cuantitativo descriptivo de corte transversal. Para la recolección de datos se utilizó la Escala de Competencia Cultural versión en español chilena, previamente validada en el contexto colombiano. La muestra estuvo conformada por 148 estudiantes de enfermería matriculados de tercer a octavo semestre, en el periodo de agosto a diciembre del 2021. Se realizó análisis univariado y bivariado mediante la prueba U de Mann Whitney y H de Kruskal Wallis.

**Resultados:**

El puntaje promedio (72.3), obtenido por los estudiantes de enfermería indica niveles moderados de competencia cultural. Se halló diferencias significativas del nivel de competencia respecto a la formación previa (p < 0.05) y a la experiencia en cuidado de pacientes con diversas culturas (p< 0.05).

**Discusión:**

Los participantes en este estudio mostraron niveles de competencia cultural similares a los encontrados en investigaciones realizadas con estudiantes de enfermería en otros países, como Corea del Sur, India, Sudáfrica, Irak, y Chile, entre otros.

**Conclusiones:**

El nivel de competencia cultural de los estudiantes de enfermería, les permite brindar acciones seguras humanas empáticas, efectivas y coherentes con la cultura de los pacientes.

## Abstract (en)

**Introduction:**

Diversity is a challenge in healthcare because it requires nurses to be culturally competent and able to provide care adapted to the beliefs, values, and practices of patients from diverse cultures.

**Objective:**

To determine the level of cultural competence of nursing students at a public university in Colombia.

**Materials and Methods:**

Quantitative, descriptive, cross-sectional study. The Chilean Spanish version of the Cultural Competence Scale, previously validated in Colombia, was used for data collection. The sample consisted of 148 nursing students enrolled from the third to the eighth semester during the period from August to December 2021. Univariate and bivariate analyses were performed using the Mann-Whitney U and the Kruskal-Wallis H tests.

**Results:**

The average score (D:72.3) obtained by the nursing students indicates moderate levels of cultural competence. Significant differences were found in the level of competence regarding previous training (p < 0.05) and experience in caring for patients from diverse cultures (p < 0.05).

**Discussion:**

Participants in this study showed levels of cultural competence similar to those found in research conducted with nursing students in other countries, such as South Korea, India, South Africa, Iraq, and Chile, among other countries.

**Conclusions:**

The level of cultural competence of nursing students enables them to provide safe, humane, empathetic, effective, and culturally coherent actions to patients.

## Resumo

**Introdujo:**

A diversidade representa um desafio na assistencia a saúde, pois supóe que os enfermeiros sejam culturalmente competentes, capazes de prestar cuidados que se adaptem as crengas, valores e práticas de pacientes de diferentes culturas.

**Objetivo:**

Determinar o nivel de competencia cultural em estudantes de enfermagem de uma Universidade Pública da Colombia

**Materiais e Métodos:**

Estudo transversal descritivo quantitativo. Para a coleta de dados, foi utilizada a versao chilena em espanhol da Escala de Competencia Cultural, previamente validada no contexto colombiano. A amostra foi composta por 148 academicos de enfermagem matriculados do terceiro ao oitavo semestre, no período de agosto a dezembro de 2021. A análise univariada e bivariada foi realizada por meio do teste Mann Whitney U e do teste Kruskal Wallis H.

**Resultados:**

A pontuagao média (D:72,3) obtida pelos estudantes de enfermagem indica níveis moderados de competencia cultural. Diferengas significativas foram encontradas no nível de competencia quanto a formagao anterior (p < 0,05) e experiencia em cuidar de pacientes com culturas diferentes (p < 0,05).

**Discussao:**

Os participantes deste estudo apresentaram níveis de competencia cultural semelhantes aos encontrados em pesquisas realizadas com estudantes de enfermagem de outros países, como Coreia do Sul, Índia, África do Sul, Iraque, Chile, entre outros.

**Conclusóes:**

O nivel de competencia cultural dos estudantes de enfermagem permite- lhes proporcionar agóes humanas seguras, eficazes, empáticas e coerentes com a cultura dos pacientes.

## Introducción

La diversidad cultural es un tema de interés en las sociedades actuales, debido a los cambios dramáticos en las cifras demográficas mundiales en las tres últimas décadas. Estos cambios, están precedidos por diversos fenómenos, entre los que se resaltan los movimientos migratorios y el reconocimiento de grupos étnicos y pueblos indígenas^1^. A esto, se suma la actual crisis migratoria o de refugiados venezolanos en Colombia, resultado de condiciones de índole político, económico, humanitario y de derechos humanos.

Estadísticas globales muestran que, para mediados del año 2020, la población de migrantes internacionales fue de 280,6 millones, lo que representa un 3,6% de la población mundial total^2^. Con respecto a las poblaciones indígenas, el International Work Group For Indigenous Affairs (IWGIA)^3^, estimó que en América latina para el año 2019, los países con mayor cantidad de población indígena fueron México, Guatemala, Bolivia, Perú, Chile y en sexto lugar Colombia con 1,5 millones de personas indígenas. Así mismo, la plataforma de Coordinación Interagencial para Refugiados y Migrantes de Venezuela (R4V)^4^, indicó que, para finales del año 2021, 6.041.690 de venezolanos se encontraban en condición de migrantes y refugiados en todo el mundo, de los cuales 4.992.215 se ubicaron en América Latina y el Caribe, con mayor proporción en países como Colombia, Perú, Ecuador, Chile y Brasil.

Colombia, ha sido reconocido como un país pluriétnico y multicultural, en consideración a la diversidad de identidades y expresiones culturales que caracterizan los pueblos y comunidades que habitan en las diferentes regiones del país. Afrocolombianos, raizales, palenqueros, rom o gitanos, pueblos indígenas, mestizos, comunidades campesinas y comunidades originadas en migraciones externas enriquecen el mosaico cultural del país^5^^,^^6^. Según el Censo Nacional de Población y Vivienda 2018 (CNPV), realizado por el Departamento Administrativo Nacional de Estadística (DANE)^7^, en Colombia la población total es de 48.258.494 habitantes, de los cuales 6.579.426 (13,7%) se autorreconoce como pertenecientes a los grupos étnicos y pueblos indígenas presentes en el país.

Con respecto al fenómeno migratorio, para el año 2020^8^, ingresaron al país 1.000.560 extranjeros, provenientes de países como Venezuela, Estados Unidos, México, Perú, Argentina, entre otros. Por otra parte, migración también reportó que, a finales de agosto de 2021, un total de 1.842.390 nacionales venezolanos residían en Colombia, de los cuales 344.688 presentaban condición de regulares, 1.182.059 se consideran en proceso de regularización y 315.643 estaban en condición irregular.

Lo anterior confirma que Colombia es una nación culturalmente diversa, lo que representa un desafío para el cuidado de la salud, así como para la formación de enfermeras, quienes deben estar dotadas de competencia cultural para atender la población considerando su pluralidad. Este concepto tiene su origen en la antropología, con Madeleine Leininger una de las pioneras en enfermería, que presenta un concepto de cuidado culturalmente congruente en su teoría de la diversidad y universalidad del cuidado^9^. Sin embargo, otros investigadores también se han centrado en la diversidad, creando varios referentes teóricos y conceptuales^10^^-^^14^.

La competencia cultural, según Burchum^15^ es un proceso continuo y no lineal, que se desarrolla a través de los atributos de conocimiento, habilidad, sensibilidad, conciencia, deseo y encuentros culturales; donde el profesional de enfermería es capaz en primer lugar de reconocer, valorar y respetar las diferencias étnicas y culturales y en segundo, de funcionar eficazmente en un contexto diverso, satisfaciendo las necesidades culturales específicas en salud que presentan los individuos y sus comunidades, con la única finalidad de brindar cuidados seguros y de calidad. Esto, sin duda alguna, impacta positivamente la respuesta que tiene el receptor de cuidado frente a su estado de salud.

Al respecto, el Consejo Internacional de Enfermería (CIE), señala que los enfermeros (as) deben ser culturalmente competentes, de forma que, puedan responder con eficacia a las necesidades culturales y lingüísticas de los individuos y comunidades. Por lo anterior, es importante que los futuros profesionales de enfermería sean formados en competencia cultural, a fin de que durante su proceso formativo adquieran los conocimientos y las habilidades para desarrollar su rol en contextos culturalmente diversos con adecuada pertinencia^16^.

Sobre los estudiantes de enfermería, investigaciones realizadas en Asia y África muestran que estos tienen rangos moderados de competencia cultural, que está asociada e influenciada por los perfiles demográficos y experiencias relacionadas con la cultura^1^. Así mismo, en otras investigaciones, los estudiantes de enfermería de Arabia Saudita, Filipinas, Turquía, Omán, India, Sudáfrica, Irak^17^ y Corea del Sur^18^ mostraron rangos moderados de competencia cultural. De modo similar, en un estudio en Arabia Saudita se determinó que la mayoría de los estudiantes de enfermería tienen niveles de competencia culturalmente consciente, lo que puede estar relacionado con la exposición a conceptos culturales, tales como la formación en cultura y el contacto con personas culturalmente diversas^19^. Por otra parte, se han reportado niveles altos de competencia cultural entre los estudiantes taiwaneses.^20^.

El objetivo del presente estudio es determinar el nivel de competencia cultural de los estudiantes de enfermería de una universidad pública de Colombia. Considerando la brecha en la producción de conocimiento sobre el tema con los estudiantes de enfermería en el país, el estudio es relevante.

## Materiales y Métodos

### Diseño y muestra

Se realizó un estudio cuantitativo, descriptivo de corte transversal. La población estuvo conformada por 239 estudiantes de enfermería matriculados de tercero a octavo semestre en el año 2021, en la Facultad Ciencias de la salud de una universidad pública en Cúcuta (Colombia), ubicada en la frontera con Venezuela, donde ha habido un crecimiento inusual de la población migrante con características culturales diversas. Se realizó un muestreo probabilístico estratificado proporcional por semestre, obteniendo una muestra de 148 estudiantes de enfermería (Tabla 1). Los matriculados de primer y segundo semestre de enfermería fueron excluidos del estudio debido a su falta de exposición a experiencias formativas tanto clínicas como comunitarias.

**Tabla 1:** Muestreo estratificado por semestre de los estudiantes de enfermería. Segundo semestre de 2021.

Semestre académico	Número de estudiantes (N)	Muestra (n) (nx= Nx / N * n)	Proporción (%)
III	50	31	21
IV	37	23	16
V	63	39	26
Semestre académico	Número de estudiantes (N)	Muestra (n) (nx= Nx / N * n)	Proporción (%)
VI	31	19	13
VII	25	16	11
VIII	33	20	13
Total	239	148	100%

Se consideraron como criterios de inclusión: 1) Estudiantes mayores de 18 años 2) Estudiantes matriculados de tercer a octavo semestre de enfermería 3) Estudiantes con experiencia práctica clínica o comunitaria 4) Estudiantes que acepten participar voluntariamente mediante la firma del consentimiento informado. Finalmente, se excluyeron estudiantes con experiencia laboral previa en el área de la salud.

### Instrumento

Un cuestionario auto administrado, estructurado en tres secciones fue utilizado para recolectar datos de los encuestados. La primera sección, conformada por 4 ítems de selección múltiple, permite recopilar información sobre algunas variables sociodemográficas como: edad, sexo, semestre académico y estado civil de los participantes. La segunda sección, consta de 5 ítem con preguntas cerradas, en las que se indaga por las experiencias relacionadas con la observación, participación y vivencia de un acontecimiento a fin con la cultura en contextos clínicos o comunitarios.

La tercera sección corresponde a la Escala de Competencia Cultural versión en inglés (CCS-S) de Perng y Watson, desarrollada en el 2012 con base en el modelo teórico de Jacqueline Burchum, en el que se define la competencia cultural, como un proceso en el que los conocimientos y habilidades se desarrollan continuamente a partir de los conocimientos, la sensibilidad y la habilidad cultural. El conocimiento cultural se describe como la adquisición constante de información sobre las diferentes culturas, que es posible a través de la formación en marcos conceptuales relacionados con el tema o la interacción cultural. La sensibilidad cultural, hace énfasis en el desarrollo del respeto, aprecio y valor hacia las diferencias culturales. Finalmente, la habilidad cultural es la destreza para comunicarse e incorporar intervenciones de cuidado adaptadas a las creencias, valores y prácticas culturales del mismo^21^.

Para el año 2018, Cruz JP et al, establecieron la versión en español de la Escala de Competencia Cultural (CCS-S), adaptándola culturalmente al idioma español en Chile. El valor del alpha de Cronbach de toda la escala fue de 0,95, indicando una excelente consistencia interna y, la fiabilidad test-retest fue de 0,85, mostrando la capacidad de medir competencia cultural consistente entre los estudiantes de enfermería. La escala está compuesta por 20 ítems, que miden habilidades individuales que pueden ser practicadas durante el cuidado de enfermería. Dichos ítems se dividen en 3 dimensiones, así: 12 afirmaciones que reflejan capacidad cultural; 6 relativas al conocimiento cultura, y 2 sobre sensibilidad cultural. Se emplea una escala Likert que puntúa de 1 a 5 (1= Totalmente en desacuerdo, 2=En desacuerdo, 3= Ni de acuerdo, ni en desacuerdo, 4=De acuerdo y 5=Totalmente de acuerdo), para calificar las respuestas, con posibles puntuaciones de 20 a 100, donde puntuaciones altas indican niveles altos de competencia cultural^22^.

Dado que la Escala de Competencia Cultural es una versión chilena que cumple con todos los requisitos de adaptación transcultural en español, para su aplicación en Colombia se realizó la adaptación cultural, que incluye la evaluación de la equivalencia sintáctica y semántica para el contexto colombiano, siguiendo la metodología del MAPI Research Institute, modificada por Aguilar^23^. Igualmente, se realizó la validez facial y de contenido. El instrumento en su adaptación a la cultura colombiana, demostró una validez y concordancia buena con 0.90 y una confiabilidad de 0.92 (Alfa de cronbach).

### Consideración ética

El protocolo del estudio recibió el aval del comité de ética en investigación de la Facultad Ciencias de la Salud de la Universidad Francisco de Paula Santander (CEIV-06-2021). Se determinaron los derechos de los participantes, dándoles plena divulgación de la naturaleza, el riesgo y los beneficios de la investigación, garantizando la protección de los sujetos durante el desarrollo de la misma.

### Recolección de datos

La recolección de datos se realizó durante los meses de octubre a diciembre de 2021. El instrumento fue enviado a la muestra estimada a través de la herramienta google forms, incluyendo consentimiento informado, objetivo del estudio y contenido de la CCS-S. El instrumento se remitió a los correos institucionales de los estudiantes matriculados de tercero a octavo semestre, en la información preliminar se indagaba sobre antecedentes de experiencia laboral previa en el área de la salud y los que respondían afirmativamente se les solicitaba no diligenciar el consentimiento ni la encuesta.

### Análisis estadístico

La información fue condensada en una base de datos del programa Microsoft Excel ® y posteriormente analizada empleando el software estadístico de ciencias sociales: SPSS versión 26. La base de datos fue almacenada en Mendeley Data^24^. El análisis estadístico se basó en la elaboración de distribuciones de frecuencia simple de las variables sociodemográficas y de experiencia académica, con su respectiva representación gráfica en diagrama de barras y sectores. Se calcularon las medidas descriptivas media, mediana, desviación estándar, valores mínimo y máximo, agrupando por intervalos las puntuaciones observadas tanto de las dimensiones como de la escala en general de competencia cultural, donde los puntos corresponden al 25, 50 y 75% de la puntuación posible para cada escala. Los resultados de las dimensiones y de la escala en general están dados en promedios, donde mayores puntajes, indican mejores niveles de competencia cultural.

Para el análisis comparativo bivariado se elaboraron tablas de contingencia entre las variables sociodemográficas, de experiencia académica y cultural y, la clasificación de la competencia cultural. El contraste de hipótesis para establecer diferencias por sexo se realizó mediante la prueba U de Mann Whitney. El contraste de hipótesis para establecer diferencias por grupos de edad y semestre académico se valoró con la prueba H de Kruskal Wallis. El nivel de significancia establecido fue 0,05.

## Resultados

### 1. Características sociodemográficas

La muestra estuvo conformada por 148 estudiantes entre los 17 y 33 años, con un promedio de 20.6 años y desviación estándar 2.2 años. Predominó el sexo femenino (81.80%); la mayoría son solteras (97.30%) y se encentraban cursando quinto semestre (26.40%), seguido de tercero (20.90%) y cuarto (15.50%). El tiempo de exposición a las experiencia clínica y comunitaria osciló entre 1 y 6 meses (66.90%).

**Tabla 2:** Caracterización sociodemográfica y experiencia académica

Variable	Categoría / Valor	n = 148
Sexo	Femenino	81,80% (121)
	Masculino	18,20% (27)
Edad	15 - 19	28,40% (42)
	20 - 24	68,20% (101)
	25 o más	3,40% (5)
Estado Civil	Soltero(a)	97,30% (144)
	Casado(a)	0,70% (1)
	Unión libre	1,40% (2)
	Separado(a)	0,70% (1)
Semestre Académico	Tercero	20,90% (31)
	Cuarto	15,50% (23)
	Quinto	26,40% (39)
	Sexto	12,80% (19)
	Séptimo	10,80% (16)
	Octavo	13,50% (20)
Duración de las experiencias	1 - 6 meses	66,90% (99)
clínicas a las que ha sido	7 - 12 meses	19,60% (29)
expuesto (meses)	13 - 18 meses	13,50% (20)

### 2. Experiencia cultural

En las experiencias culturales, sólo el 37% reportó tener formación previa en diversidad cultural, el 50.70% haber realizado cuidados a pacientes de diversas culturas y el 39.90% vivir en un entorno con diversidad cultural. Respecto a la atención en salud de grupos poblacionales especiales, predominó en el grupo de estudiantes vinculados al estudio, la atención a población de diferentes religiones (61.50%), la atención a personas en situación de discapacidad (60.10%) y a población con trastornos mentales o del comportamiento (54.70%). El 11.50% refirió no haber tenido la oportunidad de atender pacientes pertenecientes a grupos poblacionales especiales.

### 3. Competencia cultural y sus dimensiones

En la Tabla 3 se observan que en las tres dimensiones se presentó un rango moderado, con un comportamiento homogéneo en la muestra estudiada. En relación a la habilidad cultural, el promedio para esta dimensión fue 43.2 (DS: 7.6), con un coeficiente de variación del 17.6%. La dimensión conocimiento cultural, presento un promedio de 21.3 (DS: 4.1) y un coeficiente de variación de 19.6%. Finalmente, la sensibilidad cultural obtuvo un promedio de 7.9 (DS: 7.9) y un coeficiente de variación del 15.2%.

Con relación a la competencia cultural, el grupo de estudiantes vinculados al estudio exhibieron un promedio de 72.3 puntos, con desviación estándar 11.8 puntos, para un coeficiente de variación del 16.3%, observándose poca variabilidad en los resultados globales de la escala. El 49.3% de los estudiantes obtuvo una puntuación entre 51 y 75 puntos de los 100 posibles, y el 46.6% obtuvo una puntuación entre 76 y 100 puntos, lo que señala que los estudiantes tienen niveles moderados de competencia cultural.

**Tabla 3:** Medidas descriptivas para las puntuaciones observadas por subescalas y escala de competencia cultural

Medida Descriptiva	Habilidad Cultural	Conocimiento Cultural	Sensibilidad Cultural	Competencia Cul tural (Escala)
Número de ítems	12	6	2	20
Puntuación máxima posible	60	30	10	100
Media	43,2	21,3	7,9	72,3
Mediana	44	22	8	74
Moda	48	24	8	80
Desviación estándar	7,6	4,1	1,2	11,8
Mínimo	12	6	2	25
Máximo	60	30	10	100
Percentiles 25	40	19	7	66
50	44	22	8	74
75	48	24	8	80

### 4. Nivel de competencia cultural según variables sociodemográficas y experiencia cultural.

Los resultados observados en la Tabla 4, frente al nivel de competencia cultural de los estudiantes de enfermería de una universidad Pública en Colombia, señalan que no hay diferencias significativas respecto al sexo, la edad o el semestre académico (p > 0.05). Sin embargo, se observó que los hombres exhibieron mayor promedio que las mujeres.

Se logró identificar que los estudiantes entre 20 y 24 años (73,6 ± 11,6), son los que registraron mayor promedio para la escala, comparados con los estudiantes entre 15 y 19 años y de 25 o más años. Se pudo observar que el nivel de competencia cultural mejora con la experiencia universitaria, ya que los estudiantes de sexto a octavo semestre son los que obtuvieron mayor puntuación promedio para la escala, mostrando la tendencia que, a mayor semestre académico, mejor nivel de competencia cultural.

Se evidenciaron diferencias significativas del nivel de competencia cultural respecto a la formación previa en diversidad cultural (p < 0.05) y al cuidado de pacientes de diversas culturas (p < 0.05), ya que los estudiantes con dichas experiencias exhibieron mayor nivel de competencia cultural que los estudiantes que no han tenido experiencias similares. No se evidenciaron diferencias del nivel de competencia cultural en los estudiantes respecto a si viven o no en un entorno con diversidad cultural (p = 0.240).

**Tabla 4:** Nivel de competencia cultural según variables sociodemográficas y experiencia cultural.

Variable	Categorías	n	COMPETENCIAS CULTURALES Promedio ± DE	Valor p
Sexo*	Femenino	121	72,0 ± 11,5	0,384
	Masculino	27	73,7 ± 13,3	
Edad**	15 - 19	42	69,7 ± 12,4	0,091
	20 - 24	101	73,6 ± 11,6	
	25 o más	5	67,4 ± 7,1	
Semestre académico**	Tercero	31	70,5 ± 11,9	0,298
	Cuarto	23	67,3 ± 16,7	
	Quinto	39	73,5 ± 9,4	
	Sexto	19	74,1 ± 10,8	
	Séptimo	16	72,3 ± 9,1	
	Octavo	20	77,0 ± 11,1	
¿Tiene formación previa	Si	55	76,7 ± 11,0	0,000
en diversidad cultural? *	No	93	69,8 ± 11,6	
¿Ha realizado cuidados	Si	75	75,4 ± 10,1	0,005
a pacientes de diversas	No	73	69,2 ± 12,7	
culturas?*				
¿Usted vive en un entorno	Si	59	73,6 ± 10,6	0,240
con personas con diversas	No	89	71,5 ± 12,6	
culturas?*				

* Prueba U de Mann Whitney, ** Prueba H de Kruskal Wallis

## Discusión

Es importante aclarar que en el contexto de la Pandemia se suspendieron las prácticas clínicas durante dos semestres, para el primer semestre del 2021 se retomaron las prácticas con las restricciones de bioseguridad establecidas por las autoridades sanitarias. Para nivelar, en el periodo de receso académico se implementó un plan de contingencia, para recuperar prácticas clínicas y comunitarias pendientes, finalmente cuando se aplicó el instrumento los estudiantes habían podido recuperar las practicas correspondientes a 2 semestres académicos y estaban nivelando un tercer semestre, de manera que todos los estudiantes de la muestra cumplían con los criterios de inclusión.

Para este estudio, la muestra estuvo conformada predominantemente por estudiantes con edades entre los 20 y 24 años (68,20%), de sexo femenino (81,80%), de estado civil soltero (97,30%) y matriculados en quinto semestre (26,40%). Estos datos coinciden con lo reportado en Corea del Sur 18, Arabia Saudita^19^ y Chile^22^, donde el rango de edad predominante fue de 20 a 24 años (71%, 72.3%, 90.2% y 85%, respectivamente), el sexo femenino completó la mayor parte de la muestra (en orden 79.7%, 74.3%, 100% y 82.3%) y en Chile igualmente se reportó un mayor porcentaje de estudiantes solteros en Chile^22^.

Con relación a las experiencias culturales, menos de la mitad de los participantes (37%) afirmó tener formación previa o haber estado expuesto a contenidos relacionados con diversidad cultural, esta situación también se ha reportado en Chile^22^ y Arabia Saudita^19^. Llama la atención que los participantes en este estudio, han estado expuestos a contenidos culturales en los componentes curriculares disciplinares, sin embargo, como sugieren los resultados, existe un bajo reconocimiento de la formación orientada a la diversidad, lo que se evidencia en el alto número de participantes reportaron no haber recibido dicha formación.

Se esperaría que la enseñanza de contenidos culturales, a medida que avanzan el proceso de formación, aumente significativamente la competencia cultural y por tanto el estudiante desarrolle mayores capacidades para brindar cuidados óptimos, en contextos culturalmente diversos con adecuada pertinencia^25^.

Con respecto a las dimensiones: habilidad, conocimiento y sensibilidad cultural, los estudiantes de enfermería alcanzaron puntajes que corresponden a un rango moderado. Esto sugiere que, los futuros profesionales de enfermería están en el continuum intermedio para desarrollar niveles altos de competencia cultural, y en consecuencia, presentan falencias en el conocimiento sobre el aspecto cultural del cuidado, la utilización de habilidades de comunicación, la utilización de métodos para la recolección de información, la planificación de intervenciones de enfermería y la explicación de las diferencias entre las creencias y/o comportamientos de salud y los conocimientos de enfermería^26^.

En cuanto al nivel de competencia cultural, los estudiantes de enfermería del presente estudio alcanzaron un puntaje promedio de 72.3, que corresponde a un rango moderado de competencia cultural; este nivel de competencia cultural también se ha encontrado en otros estudios realizados en Finlandia^27^ y Corea del Sur^18^, solamente en Taiwan^20^ se ha reportado niveles altos de competencia cultural.

Un nivel de competencia cultural moderado indica que los estudiantes tienen la capacidad para identificar y respetar las necesidades específicas de las diversas culturas, sin embargo, muestran dificultades en su abordaje, lo que da como resultado cuidados seguros y efectivos, pero no totalmente congruentes con la cultura del paciente. En Sur C orea^18^ y Arabia Saudita^19^, se encontró que a pesar de las debilidades que pueden tener los estudiantes para brindar cuidado a pacientes provenientes de diversas culturas, estos se esfuerzan constantemente por alcanzar el nivel máximo de competencia cultural.

Los resultados de este estudio también indicaron que los estudiantes de enfermería de los semestres superiores tienen mejores puntuaciones promedio de competencia cultural, esto coincide con los hallazgos en estudiantes de enfermería en Arabia^19^ y Chile^22^, donde los estudiantes que estaban matriculados en cuarto año obtuvieron mejores niveles de competencia cultural, esto como resultado del proceso de formación en el componente de diversidad cultural y a tener experiencias culturales más amplias como resultado de las prácticas clínicas y comunitarias.

Evidentemente la competencia cultural es un proceso no lineal, en el que los conocimientos, las habilidades y la sensibilidad se van desarrollando a lo largo del tiempo y están influenciados por la formación en marcos conceptuales relacionados con el tema y por las experiencias de interacción con las diversas culturas^10^.

Los estudiantes que tenían una formación previa en diversidad y que habían experimentado el cuidado de pacientes de diversas culturas mostraron mayores puntajes promedio de competencia cultural que los que no tenían una experiencia similar. Estos hallazgos son consistentes con lo encontrado en Corea del Sur^18^, Chile^22^ y en un estudio multicéntrico realizado en 9 países^17^, en donde además de la formación y de la experiencia, se evidencio que la competencia cultural también se ve favorecida en estudiantes que residen en un entorno con personas diversas y que se han relacionado con individuos pertenecientes a grupos especiales de población.

La adquisición de competencias culturales se ve facilitada por procesos de aprendizaje curriculares y extracurriculares, que son percibidos por los estudiantes como insuficientes. Como parte del plan de estudio los contenidos teóricos complementados con prácticas clínicas que incluyen el cuidado de pacientes culturalmente diversos, proporcionan a los estudiantes de manera progresiva, conocimientos y experiencias que les permiten brindar una atención de enfermería segura y culturalmente consciente^18^. Dentro del componente extracurricular, en un estudio multicéntrico realzado en España, Bélgica, Portugal y Turquía, se destacan los intercambios académicos y estudiar con compañeros de diferentes culturas^28^.

## Conclusiones

Los estudiantes de enfermería alcanzaron puntajes promedio de habilidad, sensibilidad y conocimiento cultural, que indican rangos moderados. Esto significa que, se esfuerzan continuamente por enseñar y guiar a otros compañeros, recolectar información, entender las creencias de los pacientes y mantener un dialogo con ellos frente a las diferencias entre sus creencias, los comportamientos de salud y los conocimientos de enfermería, aspecto que probablemente está potencializado por la formación que se imparte en el programa de enfermería, vivir en un entorno con personas de diversas culturas y las experiencias culturales relacionadas con la práctica pedagógica.

La puntuación promedio para la competencia cultural ubicó a los estudiantes en un rango moderado. Lo anterior, permite afirmar que los egresados del programa de enfermería están capacitados para brindar algunas acciones de cuidados culturalmente competentes, pero no totalmente congruentes con la cultura en contextos de diversidad de acuerdo a las necesidades culturales de los individuos y comunidades. Solo se encontró diferencia estadísticamente significativa del nivel de competencia cultural con las variables formación previa y cuidado de pacientes de diversas culturas.

La perspectiva de la competencia cultural entre los estudiantes de enfermería proporcionada en este estudio, sirve como un indicador de la situación actual de la formación en diversidad cultural. A la luz de este conocimiento, las unidades académicas responsables de la formación de los futuros profesionales, deben fortalecer las estrategias pedagógicas y los contenidos teóricos y prácticos dentro del currículo, que eleven las competencias culturales en el futuro profesional de enfermería.

Se sugiere abordar este fenómeno en futuros estudios desde una perspectiva cualitativa, que permita aproximarse a las experiencias, conocimientos, actitudes y habilidades culturales desde la subjetividad de los estudiantes y profesores, logrando una comprensión integral de la competencia cultural en el enfermero en formación.
